# Molecular Identification and Intra-species Variations among *Leishmania infantum* Isolated from Human and Canine Visceral Leishmaniasis in Iran

**Published:** 2018

**Authors:** Abdolhossein DALIMI, Anita MOHAMMADIHA, Mehdi MOHEBALI, Asad MIRZAEI, Mohammadreza MAHMOUDI

**Affiliations:** 1. Dept. of Parasitology, Faculty of Medical Sciences, Tarbiat Modares University, Tehran, Iran; 2. Dept. of Medical Parasitology and Mycology, School of Public Health, Tehran University of Medical Sciences, Tehran, Iran; 3. Center for Research of Endemic Parasites of Iran (CREPI), Tehran University of Medical Sciences, Tehran, Iran; 4. Dept. of Parasitology, Faculty of Medicine, Ilam University of Medical Sciences, Ilam, Iran; 5. Dept. of Microbiology and Parasitology, Faculty of Medicine, Guilan University of Medical Sciences, Rasht, Iran

**Keywords:** Phylogeny, *Leishmania infantum*, PCR-RFLP, Sequencing, Iran

## Abstract

**Background:**

In Iran, both forms of cutaneous (CL) and visceral leishmaniasis (VL) have been re-ported; so the accurate species identification of the parasite(s) and the analysis of genetic diversity are necessary.

**Methods:**

The investigation was conducted from 2014 to 2015 in the northwest and south of Iran, where VL is endemic (7 provinces). Blood samples of patients and infected dogs were collected and sera separated for serologic examinations (DAT, rK39). Spleen or bone marrow samples from infected dogs were also collected to confirm the infection. DNAs of 70 samples amplified by targeting a partial sequence of ITS (18S rRNA–ITS1–5.8S rRNA–ITS2) gene. All the amplicons were sequenced and analyzed with restriction fragment length polymorphism (RFLP) using the TaqI enzyme.

**Results:**

The cause of all 70 VL cases, were *L. infantum*, so, the dominant specie is *L. infantum*. The sequencing results of all VL cases and RFLP analysis corroborate each other. Discrimination of Iranian *Leishmania* isolates using ITS gene gives us this opportunity to detect, identify and construct the phylogenetic relationship of Iranian isolates. In addition, detection and differentiation of *Leishmania* spp. DNA was confirmed by amplification of variable area of the minicircle kDNA (conserved sequence blocks (CSB)).

**Conclusion:**

Low divergence and high likelihood were seen among *L. infantum* isolates of human and dogs from Iran with a very slight divergence was seen between isolates from northwest and south of Iran, thus grouped in a unique clad. No correlation was observed between intraspecies divergence and geographic distribution of the isolates.

## Introduction

Visceral Leishmaniasis (VL) is an infectious systemic parasitic disease, widespread in the Old and New World. *Leishmania* parasites are obligatory intracellular protozoans of the genus *Leishmania*, which infects humans as well as domestic and wild animals, transmitted via the infective bites of Phlebotomine sand flies. Several clinical syndromes with a wide clinical spectrum of the severe disease ranges are exhibited in VL, particularly in children and immunocompromised patients (1).

VL spreads over the world in tropical and subtropical regions reported an average annual incidence of 500000 cases of the visceral form (2). In six countries: India, Bangladesh, Sudan, South Sudan, Ethiopia and Brazil, more than 90% of global VL cases occur (2, 3).

VL is caused mainly by *L. infantum* in our country, follows by splenomegaly and hepatomegaly, distributes in three foci (four provinces) in northwest and south of Iran. In a few cases, viscerotropic leishmaniasis can occur by *L. tropica*, with no specific manifestation (4, 5); and also, *L. infantum* can be a causative agent of CL (6). Dogs (*Canis familiaris*) and some wild canids such as wolves, jackals, and foxes are the main reservoir hosts for VL (7). Furthermore, VL has been reported in a sporadic form in Iran, but reports from endemic areas in northwestern and south of the country are 100–300 new cases annually (8).

In endemic areas as well as in Iran, where two or more species are the causative agent of VL, the accurate identification of the parasite(s) and the analysis of genetic diversity are necessary. Prognosis of the disease and choosing and assessing a specific chemotherapeutic regimen, effective control of the disease and avoiding the disease transmission are the main targets of these kind of the studies (9, 10).

The main molecular diagnostic technique in discriminating of *Leishmania* parasites in any kind of infected tissues is PCR-based. Different genetic targets and various post-PCR techniques provide a wide range of investigations with various gains, all over the world (11–14).

In recent years, few studies have been carried out to reveal the genetic diversity of Iranian isolates by different methods, various geographical regions for sampling and sample sizes on the reservoir or final hosts (15–17). The main aim of the present study was to assess the similarities and proximity among species responsible for the disease in the country. This can lead to national policies in order to narrow the protocols of treatments and prevention of diseases in the country.

Therefore, we have used a conventional PCR that amplifies the wide region of ITS for identification followed by sequencing and RFLP, constructed the phylogenetic tree and analyzed a number of visceral host-infecting *Leishmania* isolates from different endemic areas of Iran.

## Materials and Methods

### Study Area

**Fig. 1:**
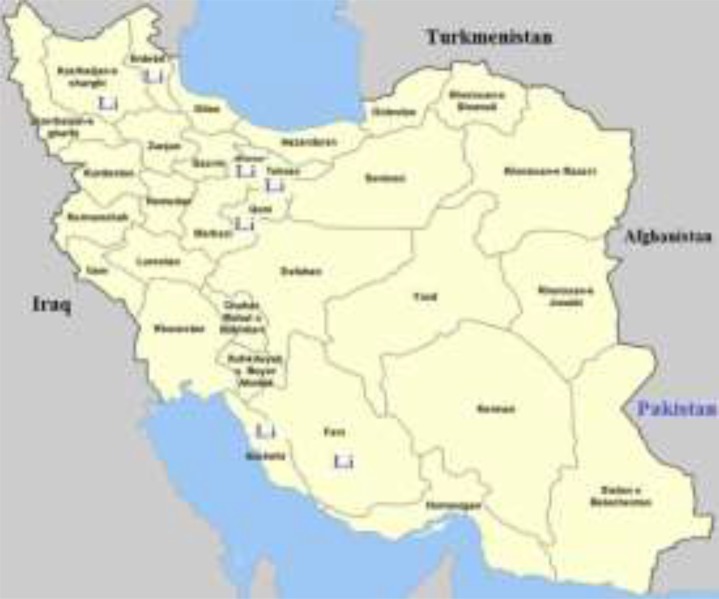
The samples were collected from 7 provinces; Fars, Boushehr, Ardabil, Alborz, East-Azerbaijan, Qom and Tehran provinces which indicated with (_Li_) in the map

The study was conducted over a couple of years (2014–2015), carried out in 7 provinces of Iran. Seventy cases with clinical manifestation of VL were enrolled in the study: 39 patients and 31 isolates from infected dogs. Fars, Boushehr, Ardabil, Alborz, East-Azerbaijan, Qom and Tehran are the provinces involved in this study (Fig. 1).

**Human:** The isolates obtained from HVL were from Ardabil Province, northwest of Iran, and the others from Tehran, Fars, Qom and Bushehr provinces.

**Dog:** Thirty-one domestic dogs selected from endemic areas from East-Azerbaijan, Ardabil, and Alborz Provinces (Table 1).

**Table 1:** List of 70 Iranian strains of *L.infantum* characterized by ITS-PCR-RFLP. Isolates were from visceral cases of leishmaniasis of Iran (2014–2015)

***No.***	***Province***	***Sample No.***	***City***	***Disease***	***Source***	***Species***
1	Fars	9	Kazeroun	HVL	Human	*L.infantum*
2	Tehran	2	Bumehen	HVL	Human	*L.infantum*
3	Boushehr	3	Borazjan	HVL	Human	*L.infantum*
4	Arbors	7	Karaj	CVL	Canine	*L.infantum*
5	Ardabil	14	Meshkin-shahr	HVL	Human	*L.infantum*
6	Qom	12	Qom	CVL	Canine	*L.infantum*
7	East-Azerbaijan	2	Ahar	HVL	Human	*L.infantum*

### Specimens

The characteristics and geographical origins of the humans (39 isolates) and dogs (31 isolates) included in this study are listed in Table 1. The sampling collection in our investigation was performed passively.

**Human:** For the passive survey, all suspected VL patients reported at least three clinical signs, including abdominal distension, paleness, and fever for at least two-week duration, were referred to local health care centers and were examined and confirmed by the physicians, were enrolled in the study. At least 3 ml blood samples were collected by trained health care workers by venipuncture into 10 ml polypropylene tubes and processed 4–10 h after collection. Approximately 2 ml of blood was used for serum separation. Blood was centrifuged at 800 gr for 5–10 min and the serums were stored at −20 °C.

**Dogs:** All the suspected CVL dogs which referred to health care centers by their owners; were examined by a veterinarian. Based on clinical evaluation (at least one clinical sign of CVL, including loss of weight, lymphadenopathy, dry exfoliative dermatitis, skin ulcers, periorbital alopecia, diffuse alopecia, or onychogryphosis) and DAT results, dogs were chosen. Spleen or bone marrow aspirations were only carried out from dogs after euthanizing or anesthesia with acepromazine or Ketamine.

Sampling was conducted after gaining accurate information about the place of infection origin. Samples and data sheets were referred to Parasitology and Entomology laboratory at the Faculty of Medical Sciences of Tarbiat Modares University.

### Parasitological Examination

Spleen/bone marrow smears were collected from dead dogs suspected of the disease from different regions of Iran, then fixed with methanol, stained with Giemsa, and examined microscopically for the presence of *Leishmania* amastigotes under high magnification (1000×) (18). Blood samples (3–5 ml) were taken from cases (humans-dogs).

**Negative and positive control:** Five human samples exhibiting no *Leishmania* antibodies (DAT^−^) and 5 dog DAT^−^ sera from non-endemic areas with no history of VL constituted negative controls. Positive control and human negative controls were prepared by the Leishmaniasis laboratory at the School of Public Health (SPH), TUMS. Canine negative controls were prepared from the Veterinary Faculty of Tehran University of Medical Sciences, TUMS. Negative and positive control and clinical samples were applied for PCR in the same condition.

### Serological Tests

All serum samples were examined by direct agglutination test (DAT) and rK39 (recombinant K39 antigen-based immunochromatographic strip test) dipstick for detection of *Leishmania*’s antibodies.

**DAT:** The Iranian strain of *L. infantum* was used for the preparation of DAT antigen in the Leishmaniasis Laboratory, at the School of Public Health and Institute of Public Health Research, Tehran University of Medical Sciences. The principal phases of the procedure for making DAT antigen were mass production of promastigotes of *L. infantum* [MCAN/IR/07/Mohebgh. (GenBank accession no. FJ555210)] (8). Peripheral blood samples from all of 70 VL cases (human and canine) were collected into tubes with sodium citrate anticoagulant 4% (Merck, Germany) for PCR testing and into tubes without anticoagulant for DAT. All samples used for serology and PCR were stored at −20 °C until use. Based on many investigations carried out in Iran, the cut-off values were determined anti-*Leishmania* antibodies titers at ≥1:320 with clinical signs considered as the disease of visceral leishmaniasis for the dogs and ≥1:3200 with clinical signs considered as *Leishmania* infection for human (8, 19).

**rK39 RDT:** For all the 70 subjects, rK39 was performed from plasma samples according to manufacturer’s instruction provided as product inserts (Cypress Diagnostic Company, Belgium). The dipsticks were briefly placed into 50μl of serum. After 1–4 min a red control line and, if positive, a second line appeared on the test field. The test is based on a combination of the protein-A colloidal gold conjugate and rK39 *Leishmania* antigen to detect anti-*Leishmania* antibody in serum or plasma. Negative controls (10 samples) were also negative by Dipstick rK39.

### Culture of Reference Strains of Leishmania

Reference strains of *Leishmania infantum* was MCAN/IR/96/LON49 that stored in liquid nitrogen. Culture was carried out in biphasic culture media (prepared from nutrient agar containing 10% whole rabbit blood overlaid with liver infusion tryptose broth containing 100–200 UI/ml penicillin G and 1 μg/ml streptomycin with 10%–20% heat-inactivated fetal bovine serum (Atlanta Biological, Atlanta, CA). The inoculated cultures were incubated at 21°C for up to six weeks and examined weekly for the presence of promastigotes. Meanwhile, Schneider Insect (HIMEDIA) and RPMI1640 (GIBCO) media were used for mass production of promastigotes.

### DNA Isolation

**Blood samples:** Prior to DNA extraction from the blood samples, 1 mL distilled water was added to 300 μL samples followed by vortexing and centrifuging at 4000 gr for 5 min, to completely remove interfering hemoglobin molecules. This step was repeated three times, and finally, the pellets were washed with PBS (14).

A noteworthy result obtained in previous studies to order to completely remove interfering hemoglobin molecules from the samples prior to DNA extraction is; washing with distilled water.

**DNA extraction:** DNA was extracted with the DNG-plus Extraction Kit (Cinnagen, Iran) according to the manufacturer’s instructions. The DNA pellet was dissolved in 50 μL of sterile distilled water and incubated in a water bath at 65 °C for 5 min. DNA concentration and quality were determined using Nanodrop ND-1000 Spectrophotometer (Nanodrop Technologies, Wilmington, DE, USA) at 260 and 280 nm. DNA samples with A260/A280 ratios between 1.8 and 2 were selected and stored at −20 °C for further analysis.

### PCR Amplification by ITS-primers

For the first amplification primers were designed based on the ITS region that identified, including forwarding primer MOF (5′-GCAGCTGGATCATTTTCCGATG-3′) and reverse primer MOR (5′-GAATTCAACTTCGCGTTGGCC-3′). The PCR product size stays between 800 and 860 bp.

### RFLP Analysis of Amplified ITS-gene.

Restriction fragment length polymorphism (RFLP) analysis was performed on the ITS amplicons which obtained from 70 blood samples and 3 the reference strains, by using the TaqI (1 μL) (Promega, USA) without prior purification. After using the restriction enzyme obtained fragments were subjected to electrophoresis in 2% agarose (Sigma-Aldrich, St. Louis, MO) at 80V in 1x TAE buffer, stained with safe stain (5 μL/100 mL), and visualized and photographed using a UV transilluminator. The obtained restricted fragments were compared with the molecular profiles of the WHO reference strains of *L. infantum* (MCAN/IR/96/LON49).

### Confirming of Identification of Leishmania Species by kDNA-snPCR

A semi-nested PCR for detection of *Leishmania* spp. DNA was performed for amplification of variable area of the minicircle kDNA (with a slight modification) (20). The combination of primers LINR4 (forward), LIN17 (reverse) and LIN19 (reverse) was used in a seminested PCR technique. These primers were designed within the conserved area of the minicircle and contained conserved sequence blocks (CSB), CSB3, CSB2, and CSB1, respectively (21). The mixture was incubated at 94 °C for 5 min followed by 30 cycles, each consisting of 30 sec at 94 °C, 30 sec at 52 °C and 1 min at 72 °C. After the last cycle, the extension was continued for a further 5 min. For the second amplification first PCR product was added to PCR-mix with 1μM, LIN19 primer for 33 cycles under the conditions as follows: 94 °C for the 30 sec, 58 °C for 30 sec and 72 °C for 1min) and the final extension at 72 °C for 10 min.

Banding patterns of *L. infantum*, *L. tropica* and *L. major* were 720, 760 and 560bp, respectively; visualized on 2% agarose gel stained with safe stain.

### Sequencing and phylogenetic Analyses

The PCR products from visceral *Leishmania* isolates (Table 1) were extracted from the gel using a Vivantis Gel Purification kit (Vivantis, Malaysia) according to the manufacturer’s protocols, were sequenced using the same forward and reverse primers used for amplification by an ABI 3730 sequencer (Bioneer, Daejeon, South Korea). The sequences were edited and manually checked with BioEdit Sequence Alignment Editor (22), aligned (data not shown) and compared with sequences from *Critidia fasciculate*, *Trypanosoma cruzi* and *Leishmania infantum*/Uzbekistan by ClustalX 2.12 (23) (http://www.clustal.org/clustal2/). The similarities among our sequences were calculated (data not shown) and phylogenetic tree (Fig. 1) was constructed by Maximum Likelihood method in Tamura 3 parameter option for DNA sequences with the complete deletion procedure, by using MEGA6 software (Molecular Evolutionary Genetic Analysis Version 6) (24). The bootstrap scores were calculated for 1000 replicates.

### Ethical Approval

The trial was reviewed and approved by the Ethics Committee of Tarbiat Modares University as well as the ethical committee of the Center for Diseases Control of Iran in accordance with the Helsinki Declaration and guidelines (Project No: 92013166). The patients and dogs’ owners were aware that their samples (blood or spleen and bone marrow) were needed for diagnosis of the disease. Physicians obtained the written the consents from the patients and dogs’ owners.

## Results

### Parasitological Results

Totally, 31 caught dogs and 3 references were checked for the presence of *Leishmania*'s parasites in the spleen or bone marrow smears by microscopy and all were positive.

### Serological Test: DAT & rK39

**DAT:** Serological responses were obtained for all 39 human sera (+ve ≥1:3200) and 31 dog sera (+ve ≥1:320) and all 70 cases were foun to be positive. All healthy patients and dogs exhibited no antibody titer with DAT.

**rK39:** Anti-*Leishmania* antibody detected in all 70 serums.

### Leishmania Identification by kDNA-snPCR and ITS-PCR-RFLP Analysis

**kDNA-snPCR:** *Leishmania* spp. kDNA were detected by seminested-PCR in 3 references. All 70 samples showed the pattern of *L. infantum*.

**Fig. 2:**
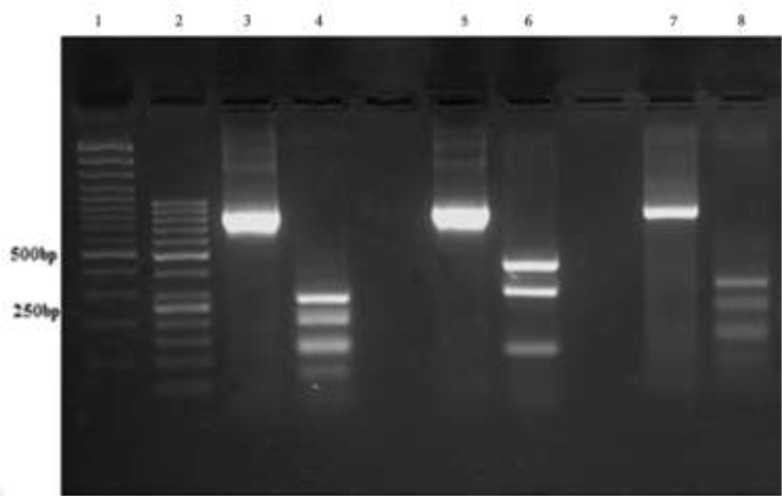
Agarose gel electrophoresis, showing PCR-RFLP results before (800–860bp) and after digestion with the restriction enzyme *Taq*I on reference strains. From left to right: Lanes 1 and 2 L: molecular weight markers (100, 50 bp). Lanes 3 and 4: *L. tropica*; lane 5 and 6: *L. major*, Lanes 7 and 8: *L. infantum*. After digestion by the restriction enzyme *Taq*I: *L. major*: 416, 296 and 141 bp.; *L. tropica*: 276, 193, 129, 115, 68 and 28 bp.; *L. infantum*: 326, 277, 142, 70 and 33 bp

**ITS-PCR-RFLP:** Amplification of ITS-rDNA from all 70 *Leishmania* isolates obtained from VL cases and 3 references, were approximately 800–860 bp. Digestion of amplicons with the Taq1 enzyme produced banding patterns, including the fragments of 326, 277, 142 and 70 bp for *L. infantum*, 416, 296, 141 and 26 bp for *L. major* and 276, 193, 129, 115, 68 and 28 bp for *L. tropica*. All samples showed the pattern of *L. infantum* (Fig. 2).

### Sequencing, Similarities and Phylogenetic Tree

**Fig. 3:**
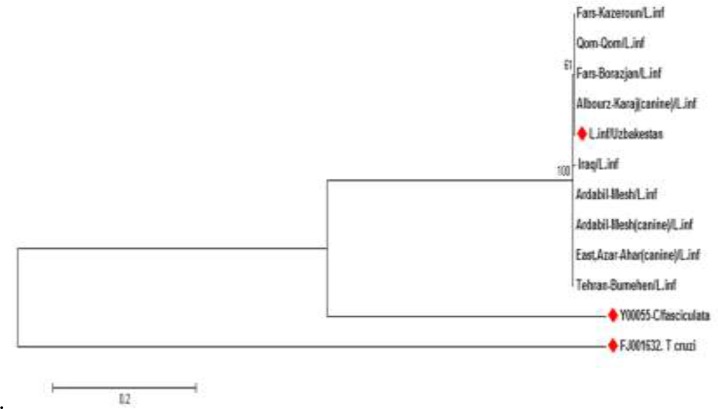
Phylogenetic tree of 8 Iranian isolates from visceral cases of leishmaniasis and 3 isolates selected from GenBank, based on ITS-gene. The tree was constructed by using the Tamura3-parameter model in MEGA software version 6. The evolutionary history was inferred using the Maximum Likelihood method, supported by 1000 bootstrap replicates. The numbers above the branches indicate the percentage of bootstrap samplings percentages. Samples isolated in the present study were compared to isolates selected from GenBank* (Red diamonds). The bar at bottom of the tree shows a scale for magnitude of genetic distance between isolates calculated by the software. *(FJ001632*/T.cruzi, C. fasciculata*/Y00055 and Uzbakestan/FN398341/*L. infantum*)

The numbers above the branches indicate the percentage of bootstrap samplings. There was no clear grouping among the 8 isolates according to their geographical origin (Fig. 3). Phylogenetic trees using Maximum Likelihood (Fig. 3) showed intra-specific variations among *L. infantum* isolates in this study and some other mentioned parasites extracted from GenBank (FJ001632/*T.cruzi* - FN398341/Uzbekistan).

Analysis of ITS sequence in our samples showed the highest (100%) and lowest similarity (97.03%) (Table 2).

**Table 2:** Levels of inter and intra-species mean similarity (one by one) among 8 Iranian of *L.infantum* Isolates obtained from visceral cases of leishmaniasis and FJ001632/*T. cruzi, C. fasciculata*/Y00055 and Uzbekistan/FN398341/*L. infantum* from GenBank, based on ITS-gene (2014–2016)

***L. infantum Isolates***	***Levels of inter and intra-species (%)***									
	***1***	***2***	***3***	***4***	***5***	***6***	***7***	***8***	***9***	***10***
1. FJ001632/*T.cruzi*	100.00	41.68	42.31	42.31	42.31	42.31	42.31	42.29	42.29	42.29
2. *C. fasciculata*/Y00055	41.68	100.00	57.01	57.01	57.01	57.01	57.01	57.72	57.72	57.72
3. Fars-Kazeroun/*L.infantum*	42.31	57.01	100.00	100.00	100.00	100.00	100.00	97.28	97.28	97.28
4. Fars-Borazjan/*L.infantum*	42.31	57.01	100.00	100.00	100.00	100.00	100.00	97.28	97.28	97.28
5. Albourz-Karaj/*L*.*infantum*	42.31	57.01	100.00	100.00	100.00	100.00	100.00	97.28	97.28	97.28
6. Qom-Qom/*L*.*infantum*	42.31	57.01	100.00	100.00	100.00	100.00	100.00	97.28	97.28	97.28
7. Uzbekistan/FN398341*/L.infantum*	42.31	57.01	100.00	100.00	100.00	100.00	100.00	97.28	97.28	97.28
8. Ardabil-MeshkinShahr/*L.infantum*	42.29	57.72	97.28	97.28	97.28	97.28	97.28	100.00	100.00	100.00
9.Tehran-Bumehen/*L.infantum*	42.29	57.72	97.28	97.28	97.28	97.28	97.28	100.00	100.00	100.00
10. East-Azerbaijan Ahar/*L.infantum*	42.29	57.72	97.28	97.28	97.28	97.28	97.28	100.00	100.00	100.00

## Discussion

Both forms of VL and CL have been already reported as important endemic diseases in Iran. Annually about 100–300 cases of VL are reported from different parts of Iran, but the actual amount is several times higher (25). The majority of the visceral cases reported from the northwest (Ardabil and East-Azerbaijan) and some of the southern provinces, including Fars and Bushehr in Iran (5) and sporadic cases have been reported from other regions (26, 27).

In Iran, DAT is routinely performed for diagnosis and seroepidemiological studies of VL, because it is simple and highly sensitive (92%–100%) (7); thus in our study, we used DAT as the standard serological test. Such passive investigations are usually encountered with the dilemma of losing samples. Asymptomatic dogs (7) and probably asymptomatic humans (28) are sources of the parasite for phlebotomine sandfly vectors and as it has been demonstrated previously asymptomatic cases reserve a high level of parasitemia in dogs and humans. On the other hand, dogs support a larger reservoir of the parasites than humans (14). In this way, the cases which did not refer to health centers were not enrolled, so we lost a significant number of isolates. Furthermore, patients who are in remission after treatment, despite having antibody titers, but there are no parasites in their bodies.

To detect *Leishmania* species in Iranian leishmaniasis focuses, conventional and molecular methods have been employed (29, 30), from different geographical areas of Iran, including small sample sizes obtained from limited geographical regions (31, 32) as well as in the large scale of sampling in a broad geographic area (16) by different molecular targets, *NAGT* gene (16) and nuclear ITS-rDNA (32).

Semi-nested PCR of kDNA, a high sensitive technique of PCR has been used formerly for detection of *Leishmania* in the sandflies (20, 33) and reservoirs (11), is used in the present study in order to confirm of species of *Leishmania* parasite which is in charge of VL in Iran.

We used ITS-RFLP and ITS-Sequencing approaches for the investigation of genetic diversity and population structure of three strains of *Leishmania* spp. from different endemic areas for VL in Iran. RFLP of (*Taq*I enzyme) ITS (18S rRNA–ITS1–5.8S rRNA– ITS2)-rDNA gene was diagnostic for *Leishmania* spp., and comparative for three *Leishmania* species (*L. tropica, L. major* and *L. infantum*) because of the size of the DNA fragment after the enzyme digestion. The electrophoretic patterns of 70 VL isolates compared with reference strains showed that all (70/70) isolates belonged to *L. infantum*. In terms of molecular epidemiology, our results are consonant with previous epidemiologic studies performed in Iran with introducing *L. infantum* as the main causative agent of VL (34).

Based on our phylogenetic tree, low divergence and high likelihood were seen among *L. infantum* isolates of human and dogs from Iran with a very slight divergence was seen between isolates from northwest and south of Iran, thus grouped in a unique clad. No correlation was observed between intraspecies divergence and geographic distribution of the isolates, as all *L. infantum* isolates from different areas of Iran causing VL with an isolate from Uzbekistan. Despite the large variations in weather conditions, geographical regions, vectors or hosts and even in reservoirs, the similarity between isolates was 97.03%–100%, so there are no noticeable differences in Iranian isolates. This is in agreement with Waki (35), which reported *L. infantum* as the less divergent complexes and in consonant with Hajjaran (16), employed ITS1 gene as DNA marker and RAPD-PCR techniques, revealed no correlation between isolates and geographical areas (36).

## Conclusion

Low divergence was seen among *L. infantum* isolates from Iran and no correlation was observed between intraspecies divergence and geographic distribution of the isolates.
